# Particulate air pollution and survival in a COPD cohort

**DOI:** 10.1186/1476-069X-7-48

**Published:** 2008-10-10

**Authors:** Antonella Zanobetti, Marie-Abele C Bind, Joel Schwartz

**Affiliations:** 1 Department of Environmental Health, Harvard School of Public Health, Boston, USA

## Abstract

**Background:**

Several studies have shown cross-sectional associations between long term exposure to particulate air pollution and survival in general population or convenience cohorts. Less is known about susceptibility, or year to year changes in exposure. We investigated whether particles were associated with survival in a cohort of persons with COPD in 34 US cities, eliminating the usual cross-sectional exposure and treating PM_10 _as a within city time varying exposure.

**Methods:**

Using hospital discharge data, we constructed a cohort of persons discharged alive with chronic obstructive pulmonary disease using Medicare data between 1985 and 1999. 12-month averages of PM_10 _were merged to the individual annual follow up in each city. We applied Cox's proportional hazard regression model in each city, with adjustment for individual risk factors.

**Results:**

We found significant associations in the survival analyses for single year and multiple lag exposures, with a hazard ratio for mortality for an increase of 10 μg/m^3 ^PM_10 _over the previous 4 years of 1.22 (95% CI: 1.17–1.27).

**Conclusion:**

Persons discharged alive for COPD have substantial mortality risks associated with exposure to particles. The risk is evident for exposure in the previous year, and higher in a 4 year distributed lag model. These risks are significantly greater than seen in time series analyses.

## Background

Worldwide studies have shown the short-term effect of particulate pollution (PM) on hospital admissions and deaths from respiratory causes [1-6].

Epidemiologic studies indicate that hospitalizations for respiratory causes are strongly related to PM exposure. Several hypotheses have been advanced for possible underlying mechanisms [7]. For example, PM may impair ventilation in COPD patients by causing airway narrowing and increasing the work of breathing [8]. In addition some particles can cause epithelial cell damage, pulmonary edema, and eventually fibrosis [9].

Particles may be deposited in the extra thoracic airways (mouth, nose, larynx), in airways of the trachiobronchial regions and in the alveolar region where the gas exchange occurs [10].

The respiratory tract deposition patterns depend on particle size and distribution within the inspired air. Biologic effects may be a function also of particle number, composition and the total surface area of the particle.

Various factors have been shown to influence particle deposition, such as age, ventilation patterns and the presence of obstructive or inflammatory airway disease [11]. Higher ventilation increases total deposition, and obstructive airway disease, such as chronic bronchitis, emphysema and asthma results in increased deposition in the lower respiratory tract [12].

Retention of particles is a function of deposition site and clearance of particles, which again may be impaired in persons with COPD. Chronic effects may also arise from recurring cycles of pulmonary injury and repair [10]. Studies have shown that high doses of particles can trigger oxidative stress and the induction of inflammation, increased blood coagulation, impaired cellular defence, and modulation of the immune system [13,14].

The effect of longer term PM exposure on survival in general cohorts has been examined in a number of studies, which mainly focused on total or cardiovascular mortality. The Harvard Six Cities prospective cohort study [15] showed an association between cardiovascular mortality and chronic exposure to air pollutants. Other studies [16,17] such as the American Cancer Society Cancer Prevention study population, most recently[17] reported an association between long term average PM and specific causes of death, such as deaths from all cardiovascular disease plus diabetes, ischemic heart disease, and dysrhythmias, heart failure, and cardiac arrest mortality.

Another cohort in seven French cities [18] found that urban air pollution was associated with increased total mortality over 25 years, and they also observed a consistent pattern for lung cancer and cardiopulmonary causes.

While these previous studies have reported an association of air pollution with survival, and sometimes cause specific survival, these have not examined whether risk changed with annual changes in exposure.

Specifically, in most published cohort studies, the exposure was taken to be long term differences in air pollution concentrations across geographic location, which does not allow examination of whether changes in annual exposure result in changes in risk. However, a recent study in Dublin [19] suggests that decreasing air pollution concentrations by a sudden intervention (a coal ban) reduced cardiovascular and respiratory deaths the next year, with no further decrease in subsequent years.

This suggests a cohort study with PM_10 _treated as a time varying covariate, with annual follow-up periods for exposure and survival, would be of considerable interest. In addition, if such studies are done within city, then by design, they eliminate the possibility of confounding by unmeasured covariates that vary across city or geography.

A recent re-analysis of the Harvard Six Cities Study, which extended the mortality follow-up period, used annual PM2.5 as a time-varying exposure, and found a significant association of PM2.5 with mortality, and moreover that most of the effect was with the previous two year's exposure [20]. However, in that study, variations in exposure both between city and between years within city contributed to the exposure gradient.

Another recent study investigated [21] whether year-to-year within city changes in annual PM exposure were associated with progression of disease or reduced survival in a study of 196,000 persons discharged alive following an acute myocardial infarction. We have applied that approach in a 34-city cohort study, with cohorts defined within each city by hospital discharge for COPD.

The focus of this study is therefore to examine the effect of exposure to particulate air pollution on persons discharged alive following an admission for chronic obstructive pulmonary disease (COPD).

## Methods

### Study Population

Using Medicare data for persons aged 65 and older for the years 1985 to 1999, we constructed a cohort of survivors with a specific condition we hypothesized might render subjects at greater risk, defining cases as an emergency admission for a primary or secondary discharge diagnosis of chronic obstructive pulmonary disease (COPD, ICD-9: 490–496, except 493); we excluded subjects who died within three months of their admission.

Medicare data provide also the date of death for those subjects who die, and therefore we could define whether they were still alive as of the end of 1999; the date of death field is derived from the Medicare enrolment database and cross-referenced with the Social Security Administration's Master Beneficiary Record [22].

Medicare provide also information on age, gender, race, number of coronary and medical intensive care days, and on factors that might modify the risk of survival, such as primary or secondary diagnoses of atrial fibrillation (ICD-9: 427.3), myocardial infarction (MI, ICD-9:410), diabetes (ICD-9: 250), congestive heart failure (CHF, ICD-9: 428), and essential hypertension (ICD-9: 401) on previous admissions, or whether these were noted as secondary diagnoses on the index admission. We defined a categorical variable for type of COPD based on ICD codes (ICD-9: 491,492,496).

Subjects alive the first of January of the year following the admission were entered into the cohort, and follow-up periods were calendar years. We excluded subjects whose death or subsequent admission occurred within the first three months of their index admission, and those who were admitted in 1999.

During the years 1985 to 1999 survival may have improved due to changes in therapy, underlying disease state, etc. To control for these changes, we used strata to allow a different underlying hazard for each 5-year interval in the study.

City characteristics as population density, percentage of population with central air conditioning, and percentage of population 65 and older with income > $50,000 were obtained from the 1990 United States census [23]; the average annual mortality rates for emphysema among people = 65 years old were obtained from the National Center for Health Statistics.

### Environmental Data

We obtained PM_10 _(particulate air matter with aerodynamic diameter less than 10 μm) data from US Environmental Protection Agency's Aerometric Information Retrieval System [24] for the years 1985 to 1999.

**Figure 1:**
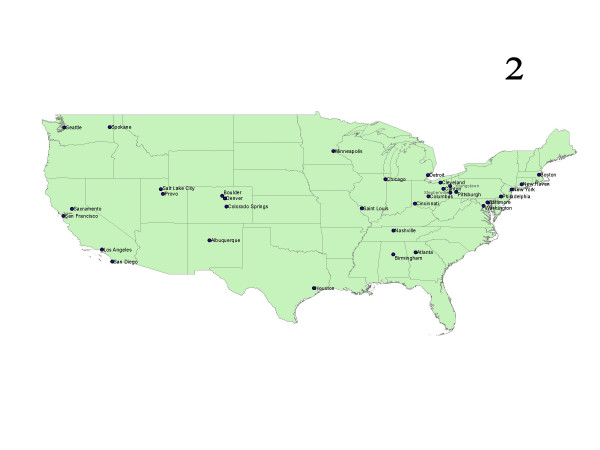
Map of the USA with the state boundaries and the cities analyzed in the study.

We selected thirty-four cities with daily monitoring of particulate matter and representing a geographic distribution across the country (Figure 1).

When more than one monitor was available in one county, PM_10 _was averaged over the county using a method previously described [25,26]. Briefly, we computed local daily mean concentrations using an algorithm that accounts for the different monitor-specific means and variances. However, before averaging, any monitor that was not well correlated with the others (r<0.8 for 2 or more monitor pairs within a community) was excluded as it likely measured a local pollution source and would not represent the general population exposure over the entire community.

For each subject and follow-up period we created yearly averages (January-December) of pollution for that year and up to the 3 previous years.

PM_10 _was then treated as a time varying covariate in the survival analysis.

### Statistical Methods

To define the cohort we assumed that each subject entered the cohort if he/she survived at least 3 months and was alive on the first January of the year following the admission. For each subject the follow up periods were 1 year periods (January – December) until the year in which they die (failure) or until December 1999 (censoring). This method has been previously described [21].

We analyzed the data with an extended Cox's proportional hazard regression model (Proc PHREG in SAS [27] which allow for time-varying covariates in survival analysis [28], as previously described [21].

We controlled for individual risk factors such as age, gender, race, season of admission, number of days of coronary and medical intensive care, previous diagnoses for atrial fibrillation and MI, and secondary or previous diagnoses for diabetes, CHF, and hypertension, time period, and season.

Season of the index discharge that defined entry into the cohort was defined as: cold (December through February), hot (June through August), and transitional. To allow for possible non-proportionality of the survival rates, time period (3 categories, one for each 5 year increment), age (5 year categories), gender, race (white, black, others), and type of COPD were treated as stratification variables.

To control for tied observations we used the appropriate likelihood function as given by Kalbfleisch and Prentice [29].

In the second stage of the analysis, the city specific results were combined using a random effect meta-regression [30]. To be conservative we report the results incorporating a random effect, whether or not there was significant heterogeneity.

We examined effect modification by city characteristics by entering them as predictor variables in the meta-regression. These included measures of socio-economic condition (percent of persons 65 and older with income > $50,000), exposure related measures (mean PM_10 _in the city), general social factors (population density), and the emphysema death rate in persons aged 65 and older as a surrogate for the smoking history of the population.

We also examined effect modification by age group (65–75 vs 76 and over), race (white vs other), and sex by including the interaction terms between the yearly PM_10 _and each variable in the model.

For each subject in each follow-up period, we considered the following possible exposure indexes: the average PM_10 _in their city in that follow-up period, and a model containing simultaneously the exposure during the follow-up period and each of the three previous years (distributed lag), to see if we could determine how the PM effect dropped off over time. We also computed the sum of the PM_10 _effect from lag 0 to the three previous years. The results are expressed as Hazard ratio (HR) for a 10 μg/m^3 ^increment of PM_10_.

## Results

Figure 1 shows a map of the USA with the state boundaries and the cities analyzed in the study.

Table 1 shows characteristics of the study population; among the 34 cities there was a higher percentage of female, and of whites, with a mean age of 76 years.

**Table 1:** Characteristics of the study population for the chronic obstructive pulmonary disease cohort, among residents of 34 US cities

	N*	**%**
**Events**	1039	100
**Deaths**	593	57.1
**Gender**		
**Male**	494	47.5
**Female**	546	52.5
**Race**		
**White**	860	82.8
**Black**	122	11.7
**Other**	57	5.5
Age**	76.4 (66.2–89.6)	
N days in coronary care**	0.35 (0–2.3)	
N days in intensive care**	0.72 (0–4.2)	
**Secondary or previous diagnoses:**		
**CHF**	205	19.7
**Diabetes**	133	12.8
**Hypertension**	287	27.6
**Previous admissions:**		
**Atrial fibrillation**	67	6.4
**MI**	38	3.7
**COPD type**		
**Chronic bronchitis**	179	13.9
**Emphysema**	108	8.4
**Chronic airway obstructions**	706	55.2

* N divided by 1000.

** Expressed as Mean (5%–95%)

During the study period there were 1,039,287 hospital discharges with COPD; of these, 57% died before the end of follow-up.

The average duration of the follow-up was 4.5 years with the range of survival times between 1 to 14 years.

The most common type of COPD was chronic airway obstructions (55%); 14% of cases had chronic bronchitis, and 8% had emphysema.

**Figure 2:**
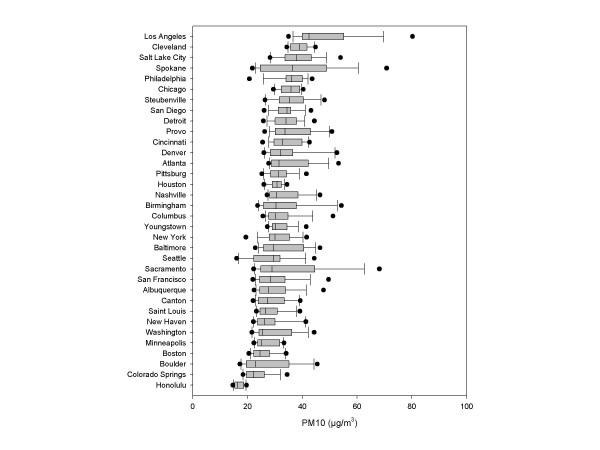
Boxplots of the of the individually assigned 1-year PM_10 _mean in each city; years 1986–1999.

Figure 2 presents the box-plots of the of the individually assigned 1-year PM_10 _mean in each city, ordered by concentration of PM_10_. Los Angeles has the highest concentrations level, while Honolulu the smallest. The average PM_10 _across all cities was 29.4 μg/m^3^.

To test the adequacy of the 5 year strata to control for secular changes in survival, we computed the annual death rate by year in the cohort (Table 2). These were higher in the first five years but changed little during the following years.

**Table 2:** Overall Mortality Rates across all cities by year

**Year**	**Mortality rates**
1986	0.20
1987	0.18
1988	0.16
1989	0.15
1990	0.14
1991	0.13
1992	0.13
1993	0.12
1994	0.12
1995	0.13
1996	0.12
1997	0.12
1998	0.12
1999	0.12

Table 3 presents the results of the PM_10 _analyses. The results are expressed as Hazard Ratio (HR) for 10 μg/m^3 ^of PM_10_. We found significant associations in the analyses adjusting for confounders, with a HR of 1.11 (95% CI: 1.06–1.15) for 10 μg/m^3 ^increase in PM_10_the same year.

**Table 3:** Hazard Ratio (HR) and 95% Confidence Interval (CI) for 10 mg/m3 increase in PM_10 _for the year of failure and for the distributed lag from the year of failure up to 3 previous years, across the 34 cities

	**HR**	**95% CI**		**P-values**
**PM_10 _annual**	1.11	1.06	1.15	0.000
				
**Distributed lag model**				
**Lag 0**	1.03	1.00	1.07	0.046
**Lag 1**	1.06	1.03	1.10	0.000
**Lag 2**	1.07	1.05	1.09	0.000
**Lag 3**	1.03	1.01	1.05	0.001
				
**Sum lags 0–3**	1.22	1.17	1.27	0.000

**Figure 3:**
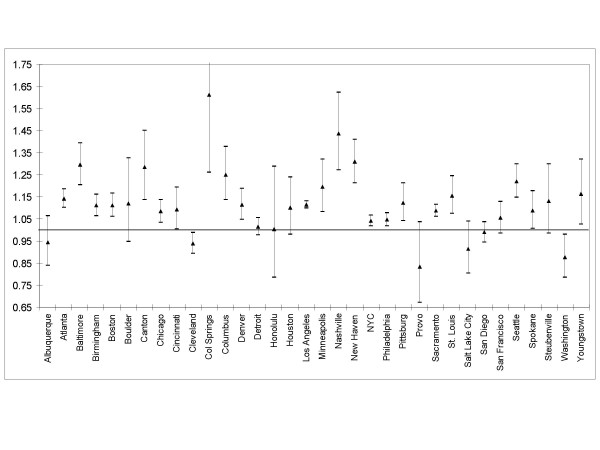
City specific results: Hazard Ratio (HR) for 10 μg/m^3 ^of PM_10_.

Figure 3 shows the city specific results for PM_10 _at lag 0. In most cities we found positive and significant associations.

When looking at the sum of the effects of PM exposure on the four years (same year and 3 preceding years) in the distributed lag model we found a hazard ratio for mortality of 1.22 (95% CI: 1.17–1.27). The PM_10 _effect sizes were higher in the distributed lag model than in the model with a single exposure period. When looking by lag, the effect on mortality risk in each year of follow-up was lower for exposure in the same year, higher for the exposure in the two previous years, and lower again for exposure three years earlier.

In the second part of the analysis we tried to explain the observed heterogeneity among cities by looking at effect modification. We used a meta-regression to examine several potential predictors. The results of these analyses are presented in Table 4. PM_10 _effect size was uncorrelated with the emphysema death rate, therefore implying that cigarette smoking did not appear to confound the association.

**Table 4:** Modification of the PM_10 _association by city characteristics across 34 US cities

**City**		**HR at the 25% Percentile**				**HR at the 75% Percentile**			
		
**Characteristics**	**P-value for modifier**	**1st Quartile**	**HR**	**95% CI**		**3rd Quartile**	**HR**	**95% CI**	
**Percent population aged 65+ in poverty status**									
	0.56	1.4	1.12	1.06	1.18	2.7	1.10	1.05	1.15
**Annual mortality rate for emphysema in 65+**									
	0.19	29.5	1.09	1.04	1.14	46.9	1.12	1.07	1.17
**PM_10 _from traffic**									
	0.27	2.3	1.12	1.07	1.18	5.0	1.09	1.05	1.14
**Air Conditioning**									
	0.77	26.0	1.11	1.06	1.17	67.0	1.10	1.04	1.16
**Population density**									
	0.09	670.0	1.13	1.08	1.18	2344.1	1.11	1.07	1.16
**Medium income**									
	0.26	4.3	1.13	1.07	1.18	5.6	1.10	1.05	1.14
**Mean summer app temperature**									
	0.91	21.6	1.11	1.06	1.16	26.3	1.10	1.05	1.16
**Variance of summer temperature**									
	0.84	11.0	1.10	1.04	1.16	21.4	1.11	1.05	1.17

Results are expressed as Hazard Ratio (HR) and 95% Confidence Interval (CI) for 10 mg/m3 increase in PM_10 _(distributed lag) estimated at the 25th percentile and the 75th percentile of the effect modifier

There was little indication of effect modification by any of the examined variables.

Finally we examined effect modification by age group (65–75 = 0 vs 76 and over = 1), race (white = 0 vs other = 1), and sex (male = 0, female = 1). The results are presented in Table 5. We found significant interactions, with a higher effect in the older age group, in non-white subjects, and in males.

**Table 5:** Hazard Ratio (HR) and 95% Confidence Interval (CI) for 10 μg/m^3 ^increase in PM_10 _for the interaction between PM_10 _and age race and sex

	**HR**	**95% CI**	
**young**	1.11	1.085	1.128
**old**	1.14	1.119	1.164
			
**female**	1.10	1.080	1.130
**male**	1.15	1.122	1.178
			
**other race**	1.23	1.194	1.271
**white**	1.12	1.099	1.141

## Discussion

We found a significant effect of long-term exposure to airborne particles on the risk of death in a large multi-city study of elderly subjects discharged alive following an admission for COPD, with a relatively large effect size compared to general population cohorts previously reported. We also found that the effect was not limited to the exposure in the each year of follow-up, with larger cumulative effects spread over the follow-up year and 3 preceding years.

This study is comparable to a similar study which analyzed admissions for myocardial infarction. That study investigated whether PM was associated with progression of disease or reduced survival in a study of 196,000 persons from 21 US cities discharged alive following an acute myocardial infarction, and found a significant effect of long term exposure to airborne particles on the risk of death, progression to heart failure and to a subsequent MI and that association persisted for several years of lag, but was falling off by lag 3 [21].

This is the first long-term study which investigated persons discharged from the hospital for COPD. One key feature of this study compared to many previously published air pollution cohort studies is that it is population based, rather than based on a convenience sample in each city. This is an important distinction. While individual exposures differ from the mean exposure in each city in each year, it seems reasonable to assume that the exposure error is Berkson, and produced no downward bias in the estimated effect [31]. That is, each person's exposure fluctuates randomly around the population average exposure, a situation that reduces power, but produces no bias in effect estimates. In contrast, for a convenience sample cohort, such as the ACS study, this is less clear, since they do not capture either the population average mortality experience in the city or the population average exposure. The ACS volunteers in city A may represent a healthier, and less exposed subset of city A then they do in city B. This, clearly, can introduce bias into the estimates.

Another key difference is the focus on shorter term, within city differences in exposure. Demonstrating that effects can be seen within a few years of exposure changes has important implications for health impact assessments. If changes in risk induced by particle exposure were permanent, we would have to wait decades to see public health improvements following changes in exposure. These results indicated that they should be seen rather within years.

The relative risks of mortality for all our four outcomes are higher than has been reported in general population studies such as the six city study (RR = 1.088, 95% CI: 1.03–1.13)[32]. This may be due to the focus on the elderly, however previous studies have not suggested such large differences in risk due to age alone, and the restriction to subjects with chronic diseases thought to increase susceptibility likely plays a role in the increased effect size. Our risks are also higher than the one observed in short term effect studies, for example our hazard ration for the same year (1.11 (95% CI: 1.06–1.15)) is higher than the previous published result of 14 US cities [6] reporting a RR of 1.02 (95% CI: 1.01–1.02) for exposure in the few days preceding a death.

Another difference between this study and previous cohort studies is the source of variation in exposure. In this study the basic analysis was conducted *within *city, and exposure variation comes from temporal changes in pollution concentration. In the other cohort studies, the source of exposure variation is across geographic area. For example the American Cancer Society study [17] contrasted covariate adjusted survival in each city with long term average pollution in that city. To the extent that geographic variations in unmeasured confounders such as diet, usual treatment for medical conditions, etc exist as potential confounders to those studies, that problem is obviated in our study design. Instead, we must contend with unmeasured temporal confounding. We have used separate strata within each city for 5 year intervals to capture such changes, but as with any study, this may be imperfect control. Nevertheless, the finding of an association between longer term exposure to airborne particles and survival in a study with such different susceptibilities to confounding adds considerable strength to the evidence of a much larger effect of longer term exposure to particles on mortality risk.

The use of longitudinal rather than cross-sectional exposure gradients in this study may also explain some of the differences in effect size estimates, as the variation in central station monitoring and personal exposure over time may be more correlated than similar variations over space. In addition, the American Cancer Society study used monitors within the multi-counties metropolitan areas to assign exposure, whereas our subjects are generally matched to monitors in the same city or county. A recent re-analysis of the American Cancer Society study restricted to subjects living in the same county as the monitor reported a higher relative risk [33].

This study presents some limitations; one is that Medicare does not provide the underlying cause of death, limiting our ability to study susceptibility.

The main limitation, however, is the absence of information on subject characteristics such as smoking, body mass index, or medicine use. In our model we controlled for all the available personal characteristics such as age, race, gender, severity of the index admission, and detailed data on previous and secondary diagnosis. Moreover, we conducted a city-specific analysis to remove location-specific differences from the analyses. Hence differences across cities in smoking rates, etc cannot confound the association, as only the temporal variability in pollution within city contributes to the association. We do not believe year to year changes in PM_10 _concentrations are correlated with year to year changes in smoking rates within city, but we cannot definitively exclude the possibility. Further, the emphysema death rate in persons aged 65 and older, which is strongly associated with smoking history, did not modify the PM associated risk.

## Conclusion

Our findings suggest that long-term exposure to particulate matter elevates the risk of mortality in susceptible population defined by COPD admissions. The finding that the effect seems increasing to the preceding two years of exposure has important implications for public health. It indicates that reductions in air pollution should be followed quickly by improvements in public health, rather than the decades some have suggested. This is in accordance with some other recent studies [20,21,34], and heightens the urgency for such measures.

## Competing interests

The authors declare that they have no competing interests.

## Authors' contributions

AZ participated in the design of the study, performed the statistical analysis, and drafted the manuscript. MACB prepared the datasets, performed the statistical analysis. JS participated in the design of the study, and helped writing the manuscript, revising it critically for important intellectual content. All authors read and approved the final manuscript.
